# Efficacy of radiation plus transarterial chemoembolization and lenvatinib in hepatocellular carcinoma with portal vein tumor thrombus

**DOI:** 10.3389/fonc.2023.1320818

**Published:** 2023-12-19

**Authors:** Aoran Dong, Meiyan Zhu, Zeyu Zhang, Wenzhe Fan, Zhiqiang Wu, Yong Chen, Jianfei Tu, Yaojun Zhang, Wenquan Zhuang, Xiaofang He, Zhenwei Peng

**Affiliations:** ^1^ Department of Radiation Oncology, The First Affiliated Hospital of Sun Yat-sen University, Guangzhou, China; ^2^ Department of Interventional Oncology, The First Affiliated Hospital of Sun Yat-Sen University, Guangzhou, China; ^3^ Department of Interventional Radiology, The First Affiliated Hospital of Sun Yat-sen University, Guangzhou, China; ^4^ Key Laboratory of Imaging Diagnosis and Minimally Invasive Intervention Research, Lishui Hospital of Zhejiang University, Lishui, China; ^5^ Department of Hepatobiliary Oncology, Sun Yat-sen University Cancer Center, Guangzhou, China; ^6^ Clinical Trials Unit, The First Affiliated Hospital of Sun Yat-sen University, Guangzhou, China

**Keywords:** hepatocellular carcinoma, portal vein tumor thrombus, external beam radiation, TACE, lenvatinib

## Abstract

**Background:**

We aimed to investigate the efficacy of a novel regimen, external beam radiation (RT) combined with trans arterial chemoembolization (TACE) and lenvatinib (LEN), in the treatment of hepatocellular carcinoma (HCC) with portal vein tumor thrombus.

**Methods:**

We prospectively observed 102 participants from three tertiary medical centers in China between October 2018 and October 2020, who chose either RT plus TACE and LEN (RT-TACE-LEN) or TACE and LEN (TACE-LEN). LEN (12 mg or 8 mg daily) was administrated orally and continued until progression or intolerable side effects were noted. TACE was given one day after administration of LEN, and RT began within 4 weeks after the first TACE. The median dose/fraction of RT was 50 Gy/25 fractions (range: 45-60 Gy/25 fractions). Overall survival and progression free survival were compared between two groups, and complications were assessed.

**Results:**

Both 51 patients received RT-TACE-LEN and TACE-LEN, respectively. Most patients had tumor size> 5 cm (73.8%) and tumor number≥ 2 (69.9%). The overall incidence of toxicities was significantly higher in RT-TACE-LEN group than TACE-LEN group (100% *vs.* 64.7%, *p*< 0.001), but incidences of grade 3-4 toxicities were comparable (54.9% *vs.* 49.0%, *p*= 0.552). Both median overall survival (22.8 *vs.* 17.1 months, *p*= 0.031) and median progression-free survival (12.8 *vs.* 10.5 months, *p*= 0.035) were significantly longer after RT-TACE-LEN treatment than TACE-LEN.

**Conclusions:**

The addition of RT to TACE and LEN was safe, and might improve clinical outcomes of patients with advanced HCC, which needs conformation from further studies.

## 1 Introduction

Hepatocellular carcinoma (HCC) is one of the most aggressive malignant tumors, leading to the third leading cause of cancer death worldwide (1, 2). More than 60% of patients are diagnosed at an advanced stage presenting with portal vein tumor thrombus (PVTT) (3, 4), which is a critical prognostic factor contributing to poor survival outcomes (5–7). Currently, systemic treatments including atezolizumab plus bevacizumab, sorafenib and lenvatinib (LEN), are recommended as first-line treatments for advanced HCC (8–11), However, patients with PVTT derive limited benefit from the current treatments and require a systemic-locoregional combination therapy to improve efficacy (11–13). In the recent years, locoregional treatments including trans arterial chemoembolization (TACE) and radiotherapy have showed promising survival outcomes in treating patients with PVTT (14–16).

TACE is one of the most commonly used local treatments for HCC (17), and is the recommended standard of care in the treatment of intermediate HCC charactered by large or multinodular intrahepatic tumors (18). A recently-published phase III randomized controlled clinical trial (LAUNCH) demonstrated clinically meaningful and statistically significant improvements in overall survival (OS) and progression-free survival (PFS) with LEN plus TACE over LEN in patients with advanced HCC (19), which may be a potential first-line treatment for advanced HCC. However, presence of PVTT is closely correlated to internal and external liver metastasis and ultimately leads to treatment failure (20, 21). Efficacy of the current therapies, including combination therapy of TACE and LEN, is far from satisfaction in the treatment of PVTT (21). Therefore, developing novel combination therapies that are effective against advanced HCC with PVTT is still under great need.

During the past few years, external beam radiation (RT) has been increasingly applied in the treatment of PVTT, reporting significant survival benefits with objective response rates ranged from 45-60% and median OS ranged from 10-19.5 months (22, 23). Previous studies have demonstrated that combination therapy of RT and other local or systemic treatments including TACE and sorafenib, could achieve significantly better clinical survival outcomes than monotherapy in advanced HCC with PVTT (24–26), which indicated RT as an optimal option in the consideration of efficient combination treatment modality for HCC with PVTT. However, the safety and efficacy of adding RT to TACE and LEN in the treatment of HCC with PVTT has not been illustrated so far.

Therefore, we conducted this study to estimate the safety and efficacy of RT plus TACE and LEN in HCC patients with PVTT, aiming to provide clinical evidence for this novel regimen in the treatment of advanced HCC.

## 2 Methods

### 2.1 Study design and participants

This prospective observation study was conducted from October 2018 to October 2020 in three tertiary medical centers in China, including the First Affiliated Hospital of Sun Yat-sen University, Sun Yat-sen University Cancer Center and Lishui Hospital of Zhejiang University. It was approved by the institution’s Ethics Committee (approval number: [2020]247) and conformed to the standards of the Declaration of Helsinki. Before enrollment, all the participants provided written informed consent comprising a data privacy item for data collection and analysis for research purposes.

Patients with HCC showing PVTT were eligible for inclusion if they had the following characteristics: (a) age 18-75 years; (b) at least one measurable intrahepatic lesion on the basis of modified Response Evaluation Criteria in Solid Tumors (mRECIST); (c) an intrahepatic lesion consisting of a single tumor (≤ 10.0 cm) or multiple tumors (≤ 3 foci) with the tumor burden< 50%; (d) Eastern Cooperative Oncology Group (ECOG) performance status score of 0 or 1; (e) Child-Pugh class A or B7; (f) life expectancy of at least 3 months; and (g) satisfactory blood, liver, and kidney function parameters. The acceptable blood, liver, and kidney parameters were (i) neutrophil count≥ 1.5 × 10^9^/L; (ii) platelet count≥ 60 × 10^9^/L; (iii) hemoglobin concentration≥ 90 g/L; (iv) serum albumin concentration≥ 30 g/L; (v) bilirubin≤ 50 μmol/L; (vi) aspartate aminotransferase and alanine aminotransferase< 5 × upper limit of normal and alkaline phosphatase< 4 × upper limit of normal; (vii) extended prothrombin time< 6 seconds of upper limit of normal; and (viii) serum creatinine< 1.5 × upper limit of normal.

Exclusion criteria included: (a) history of liver and adjacent tissue radiation; (b) medical history of hepatic decompensation, such as hepatic encephalopathy and esophageal or gastric variceal bleeding; (c) extrahepatic spread; (d) combination with other malignant diseases; (e) contraindications for TACE; (f) pregnant and lactating women; (g) severe dysfunction of the heart, kidney, or other organs.

### 2.2 Treatment protocol

#### 2.2.1 Treatment schema

Treatment allocation for each patient was decided by a multi-disciplinary team consisted of hepatologist, interventional radiologists, radiation oncologists and liver surgeon, as well as patient’s request. Treatment schema was summarized in **Supplementary Figure 1**. In both groups, LEN was initially provided to patients first, and then TACE was given one day after administration of LEN. On-demand TACE was performed if there was incomplete necrosis or tumor regrowth based on imaging evaluation. In the RT-TACE-LEN group, RT began within 4 weeks after the first TACE.

#### 2.2.2 Lenvatinib administration

LEN (Lenvima; Eisai Co., Ltd., Tokyo, Japan) was administered orally based on the prescribing information, at a standard dose of 12 mg (for body weight≥ 60 kg) or 8 mg (for body weight< 60 kg) daily. Dose reductions were permitted according to drug-related toxicity grade as recommended. LEN treatment would be terminated if there was disease progression or unacceptable toxicity during treatment. LEN was continuously administered without interruption during TACE and RT.

#### 2.2.3 TACE

The choice of conventional TACE or drug-eluting beads TACE was determined by consensus between interventional radiologists and hepatologists, and each patient was required to use the same chemoembolization agent throughout the study duration. Procedures were performed as previously recommended (27). Briefly, conventional trans arterial chemoembolization (cTACE) was performed with a maximum dose of 50 mg and 8 mL epirubicin and Lipiodol (Guerbet, Pairs, France), respectively. Epirubicin was infused into the selective catheterization of the feeding artery as the chemotherapeutic agent. The feeding arteries were then embolized using an emulsion of epirubicin and iodized oil mixture, followed by an absorbable gelatin sponge (Gelpart: NipponKayaku, Tokyo, Japan). Drug-eluting transcatheter arterial chemoembolization (DEB-TACE) was performed with 100-300 μm DC beads (BTG, London, UK) loaded with 50 mg of epirubicin or 50-100 μm Hepasphere (Nippon-Kayaku, Tokyo, Japan) loaded with 50 mg of fine powder cisplatin (IA-call; Nippon-Kayaku). Embolization was performed until stasis in the tumor feeding vessels, preserving flow in the segmental and lobar arteries. Thereafter, TACE was repeated every 6-8 weeks at the discretion of the investigators.

#### 2.2.4 External beam radiation

External beam radiation therapy was delivered using an intensity-modulated radiation therapy technique and carried out using 6-MV X-ray with a linear accelerator (Varian Medical Systems). Gross target volume was defined as intrahepatic tumors and vascular invasion, referring to the CT, MRI, and angiography findings. The clinical target volume involved a 5 mm margin around the gross target volume. Planning target volume was expanded to include a 5mm margin from the clinical target volume. The treatment goal was 45-60 Gy in 25 fractions to the isocenter of the planning target volume. The prescription dose was adjusted based on proximity to the at-risk organ. RT was continued until completion or unacceptable toxicity.

### 2.3 Treatment response and safety analyses

The participants were evaluated by physical examination, monitoring of symptoms and toxicity weekly during RT, and after each TACE. Contrast-enhanced dynamic CT or MRI was performed 6-8 weeks after each TACE and one month after the completion of RT to assess tumor response. Thereafter, patients received CT or MRI examination every 3 months at follow-up. Two independent radiologists assessed tumor response blindly according to the mRECIST (28), and any inconsistency of assessment results was resolved by discussion. The short-term efficacy was evaluated based on the tumor response at 3 months after the completion of the last TACE in each group, or at 3 months after the completion of RT if no TACE was performed after RT. The primary endpoint of the current study was OS and the secondary outcome included PFS, objective response rate (DCR), objective response rate (ORR) and safety. OS was defined as the interval between the date of initial treatment and the date of death or last follow-up. PFS was defined as the date of initial treatment and the date of progression, death, or last follow-up. The ORR was defined as the proportion of participants with a complete response (CR) or partial response (PR), and the DCR was defined as the proportion of participants with CR, PR, or stable disease (SD). Treatment-related toxicity was graded according to the Common Terminology Criteria for Adverse Events (ver. 5.0). The follow-up was censored on December 31, 2022.

### 2.4 Statistical analysis

The two groups were compared by using Student’s *t* test for continuous data and Chi-square test for categorical data. Stratified Cox proportional hazards models were used for univariable and multivariable analyses. To mitigate selection bias and minimize the potential influence of confounding factors, we employed a propensity score matching (PSM) analysis, wherein we matched patients who received TACE-LEN or RT-TACE-LEN treatments. Baseline variables demonstrating p-values of less than 0.2 in both groups were included in the PSM model for the calculation of propensity scores. These variables encompassed age, tumor size, tumor number, presence of portal vein tumor thrombosis (PVTT), Eastern Cooperative Oncology Group (ECOG) performance status, Child-Pugh class, α-fetoprotein levels, and Hepatitis B/C virus infection. For the matching process, HCC patients were paired in a one-to-one ratio using logistic regression based on their propensity scores. Survival analysis was estimated by the Kaplan-Meier method, and differences between groups were assessed by log-rank test. A 2-tailed p value of < 0.05 was considered statistically significant. All statistical analyses were performed using SPSS, version 22.0 (SPSS Inc., Chicago, IL).

## 3 Results

### 3.1 Baseline characteristics

**Figure 1:**
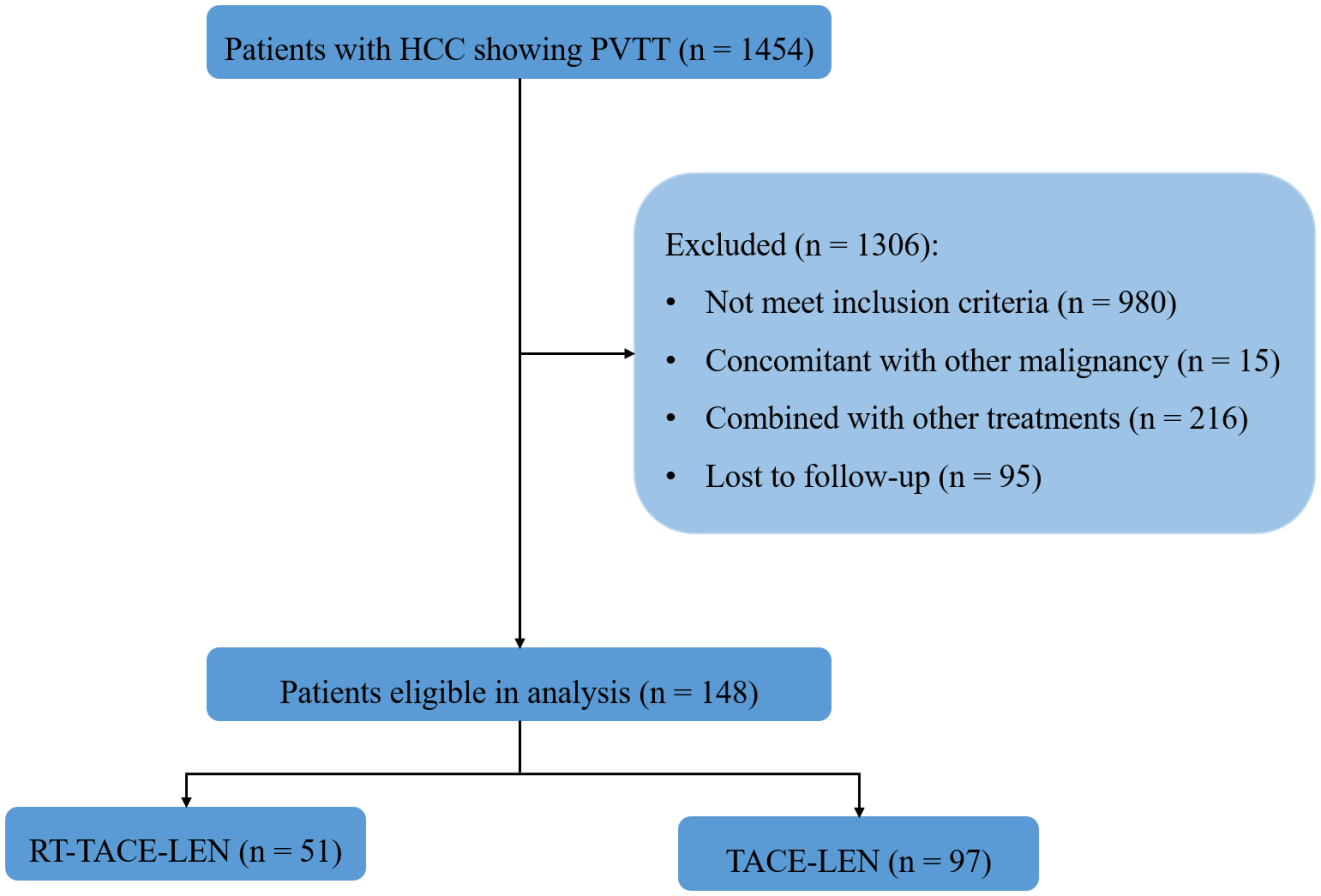
Patient flow diagram.

From October 2018 to October 2020 (**Figure 1**), 148 eligible participants were diagnosed, among which 51 participants received RT-TACE-LEN and 97 participants received TACE-LEN. All included patients were staged as BCLC C stage. After 1:1 PSM, two matched groups were derived including 51 participants in the RT-TACE-LEN group and 51 participants in the TACE-LEN group. The baseline characteristics of the two matched groups are described in **Table 1**. No significant difference was found in any variables between these two groups (all *p*> 0.05). Most patients had tumor size> 5 cm (73.8%) and tumor number≥ 2 (69.9%). According to the Cheng’s classification (5), 42.7% patients were classified as I-II grade of PVTT and the rest were III-IV grade. The most common HCC etiology was chronic HBV infection (81.6%). Majority of patients presented with well-compensated liver function (Child-Pugh class A, 80.6%) and good ECOG performance status (ECOG performance status= 0, 88.3%). The median dose/fraction was 50 Gy/25 fractions (range: 45-60 Gy/25 fractions). 50 Gy with 25 fractions of 2 Gy per fraction was the most common dose regimen (18 of 51 patients, 35.3%), followed by 45 Gy in 25 fractions of 1.8 Gy (16 of 51 patients, 31.4%), 55 Gy in 25 fractions of 2.2 Gy (13 of 51 patients, 25.5%), and 60 Gy in 25 fractions of 2.4 Gy (3 of 51 patients, 5.9%). Altogether, 85 sessions of DEB-TACE and 80 sessions of cTACE were carried out in the RT-TACE-LEN group, while 86 and 84 in the TACE-LEN group, respectively. The mean numbers of TACE sessions for each patient were both 2 in these two groups (**Supplementary Table 2**).

**Table 1:** Baseline characteristics of hepatocellular carcinoma patients with PVTT after propensity score matching.

Variable	Patients, No. (%)			*p* value^a^
	Total (*n* = 102)	RT-TACE-LEN (*n*= 51)	TACE-LEN (*n*= 51)	
Age, median (range), year	53.5 (18–75)	54 (18–75)	53 (27–75)	0.507
Gender				0.136
Male	94 (91.3)	46 (90.2)	48 (94.1)	
Female	8 (7.8)	5 (9.8)	3 (5.9)	
Tumor size, cm				0.650
≤ 5	26 (25.2)	14 (27.5)	12 (23.5)	
> 5	76 (73.8)	37 (72.5)	39 (76.5)	
Tumor, No.				0.385
1	30 (29.1)	17 (33.3)	13 (25.5)	
≥ 2	72 (69.9)	34 (66.7)	38 (74.5)	
Portal vein tumor invasion				0.110
I-II grade	44 (42.7)	26 (51.0)	18 (35.5)	
III-IV grade	58 (56.3)	25 (49.0)	33 (64.7)	
ECOG performance status				0.750
0	91 (88.3)	45 (88.2)	46 (90.2)	
1	11 (10.7)	6 (11.8)	5 (9.8)	
Child-Pugh class				0.799
A	83 (80.6)	42 (82.4)	41 (80.4)	
B	19 (18.4)	9 (17.6)	10 (19.6)	
α-Fetoprotein, ug/L				0.692
≤ 400	54 (52.4)	28 (54.9)	26 (51.0)	
> 400	48 (46.6)	23 (45.1)	25 (49.0)	
Hepatitis B virus infection				0.603
Negative	18 (17.5)	10 (19.6)	8 (15.7)	
Positive	84 (81.6)	41 (80.4)	43 (84.3)	
Hepatitis C virus infection				> 0.999
Negative	97 (94.2)	48 (94.1)	49 (96.1)	
Positive	5 (4.9)	3 (5.9)	2 (3.9)	

a Categorical variables were estimated by Chi-square test, and continuous variables were estimated by Student’s t test.

RT, radiation; TACE, trans arterial chemoembolization; LEN, lenvatinib; No., number; ECOG, Eastern Cooperative Oncology Group.

### 3.2 Safety

Any-grade toxicities were noted in 51 (100%) and 33 (64.7%) patients in the RT-TACE-LEN group and the TACE-LEN group, respectively (**Table 2**). Although the overall incidence of toxicities of any grade was higher in RT-TACE-LEN compared with TACE-LEN (*p*< 0.001), the rates of grade 3-4 toxicities were comparable (54.9% *vs.* 49.0%, *p*= 0.552). Toxicity profiles showed no significant difference between these two groups. Elevated aspartate aminotransferase was the most common grade 3-4 toxicity in both RT-TACE-LEN (*n*= 10, 19.6%) and TACE-LEN (*n*= 9, 17.6%) groups, following by elevated alanine aminotransferase (15.7% *vs.* 15.7%) and Hyperbilirubinemia (11.8% *vs.* 9.8%) All these toxicities were treated expectantly and recovered or restored to grade 1-2 post-treatment.

**Table 2:** Acute toxicity in patients treated by RT-TACE-LEN or TACE-LEN.

Toxicity	Any Grade		*p* value	Grade 3-4		*p* value
	Group, No. (%)			Group, No. (%)		
	RT-TACE-LEN (*n*= 51)	TACE-LEN (*n*= 51)		RT-TACE-LEN (n= 51)	TACE-LEN (n= 51)	
Ascites	7 (13.7)	6 (11.8)	0.767	3 (5.9)	2 (3.9)	0.645
Elevated ALT	12 (23.5)	10 (19.6)	0.630	8 (15.7)	8 (15.7)	>.999
Elevated AST	13 (25.5)	11 (21.6)	0.641	10 (19.6)	9 (17.6)	0.799
Hyperbilirubinemia	10 (19.6)	9 (17.6)	0.799	6 (11.8)	5 (9.8)	0.750
Hypoalbuminemia	9 (17.6)	7 (13.7)	0.586	1 (2.0)	1 (2.0)	>0.999
Total	51 (100.0)	33 (64.7)	< 0.001	28 (54.9)	25 (49.0)	0.552

ALT, alanine aminotransferase; AST, aspartate aminotransferase; RT, radiation; TACE, trans arterial chemoembolization; LEN, lenvatinib.

### 3.3 Efficacy analysis

The median follow-up for all patients was 24.0 months (range: 20.0-25.6 months). By the time of assessment of the short-term efficacy, 2 patients achieved CR and 27 patients achieved PR after treatment in the RT-TACE-LEN group, with an ORR of 56.9% and a DCR of 92.1%. As for TACE-LEN group, the overall frequencies of CR (2.0%, 1/51) and PR (49.0%, 25/51) were comparable to those of RT-TACE-LEN group, with an ORR of 51.0% and a DCR of 90.2% (**Supplementary Table 1**).

**Figure 2:**
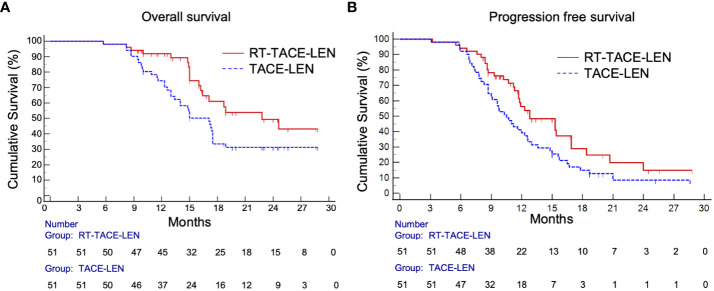
Kaplan-Meier curves show **(A)** progression-free survival (*p*= 0.035) and **(B)** overall survival (*p*= 0.031) in the RT-TACE-LEN group and TACE-LEN group.

As for survival outcomes, the 1-year and 2-year PFS rates were 56.2% and 14.9% in the RT-TACE-LEN group, respectively, which were higher than those in the TACE-LEN group (39.2% and 8.5%, respectively). Patients in the RT-TACE-LEN group had a significantly longer median PFS than those in TACE-LEN group (12.8 *vs.* 10.5 months, *p*= 0.035; **Figure 2A**). As for OS, patients in the RT-TACE-LEN group showed higher 1-year and 2-year OS rates than patients in the TACE-LEN group (1-year OS rate: 91.8% *vs.* 74.4%; 2-year OS rate: 48.1% *vs.* 31.3%). And RT-TACE-LEN group presented with a significantly longer median OS than TACE-LEN as well (22.8 *vs.* 17.1 months, *p*= 0.031; **Figure 2B**).

### 3.4 Uni- and multivariate analyses

In the univariable analysis, treatment allocation with RT-TACE-LEN was a significantly favorable factor for both PFS and OS, while older age, gender of male, lager tumor size, tumor number of more than 1, higher grade of PVTT, Child-Pugh class A, higher AFP level, Hepatitis B virus infection and Hepatitis C virus infection showed no statistical difference (**Table 3**). Multivariable analysis demonstrated that RT-TACE-LEN treatment was associated with better PFS (HR 0.577; 95% CI 0.364-0.915; *p*= 0.019) and OS (HR 0.529; 95% CI 0.298-0.938; *p*= 0.029) over TACE-LEN treatment, while ECOG performance status= 0 only contributed to better PFS (HR 0.397; 95% CI 0.160-0.989; *p*= 0.047) but not OS (HR 0.387; 95% CI 0.120-1.248; *p*= 0.112).

**Table 3:** Univariate and multivariate analyses of predictors of overall survival and progression free survival after treatment.

Variable	Overall survival				Progression free survival				
	Univariate analysis	Multivariate analysis			Univariate analysis	Multivariate analysis			
	*p* value	Hazard Ratio	95% CI	*p* value	*p* value	Hazard Ratio	95% CI	*p* value	
Age (≤ 60/> 60yrs)	0.288				0.182				
Gender (Male/Female)	0.432				0.423				
Tumor size (≤ 5/> 5cm)	0.735				0.178				
Tumor number (1/≥2)	0.898				0.244				
Portal vein tumor invasion (I-II/III-IV)	0.567				0.204				
ECOG performance status (0/1)	0.109	0.387	0.120-1.248	0.112	0.027	0.397	0.160-0.989	0.047	
Child-Pugh class(A/B)	0.505				0.501				
α-Fetoprotein (≤ 400/> 400 ug/L)	0.834				0.915				
Hepatitis B virus infection (Positive/Negative)	0.262				0.403				
Hepatitis C virus infection (Positive/Negative)	0.216				0.999				
Treatment allocation (RT-TACE-LEN/TACE-LEN)	0.002	0.529	0.298-0.938	0.029	0.002	0.577	0.364-0.915	0.019	

CI, confidence interval; ECOG, Eastern Cooperative Oncology Group; RT, radiation; TACE, trans arterial chemoembolization; LEN, Lenvatinib.

### 3.5 Subsequent treatments after progression

Ultimately, there were 30 and 45 patients undergoing progression after treatment in RT-TACE-LEN group and TACE-LEN group, respectively (**Supplementary Table 3**). Thirteen patients in the RT-TACE-LEN group received anti-PD-1 immunotherapy and 10 patients received other targeted therapy except LEN after progression, while the numbers of patients in the TACE-LEN group were, respectively, 18 and 16. The numbers of patients receiving TACE, radiotherapy or systemic chemotherapy after progression were all less than 3 in both groups.

## 4 Discussion

Currently, atezolizumab plus bevacizumab, sorafenib and lenvatinib are recommended as standard of care for patients with advanced HCC. Though bevacizumab/azolizumab is superior to sorafenib according to the results of IMBRAVE-150 published in 2020 (11), sorafenib and LEN, which are similar in oncologic outcomes, still play a critical role in treating advanced HCC, especially for patients diagnosed with HCC before 2020. In our study, eligible patients were recruited from October 2018 to October 2020, when LEN was recommended as first-line treatment for advanced HCC. In addition, LEN presented much better ORR than sorafenib, though they showed similar survival outcomes (13). Therefore, both groups of patients received LEN in the current study. To the best of our knowledge, this is the first clinical trial to estimate the safety and efficacy of RT plus TACE and LEN in HCC with PVTT. Our study demonstrated that RT-TACE-LEN favored significantly longer PFS and OS over TACE-LEN, and the therapeutic modality was an independent prognostic factor for both PFS and OS. Besides, treatment-related toxicities of RT-TACE-LEN were acceptable and manageable.

PVTT was an important prognostic factor for poor survival outcomes among patients with HCC. It not only promotes intrahepatic tumor spread but also rapidly decreases blood supply to the liver, causing rapid deterioration of liver function and increased risk of portal hypertensive complications (29), which greatly limits the application of TACE in the treatment of PVTT. As for patients with main PVTT, the median OS after TACE treatment was only 5 months (16). So far, there have been no worldwide consensuses or guidelines on the treatment of HCC with PVTT. Western guidelines regard PVTT as an advanced stage of HCC with little hope for a cure, and thus only recommend sorafenib or LEN monotherapy as standard of care (8, 30), resulting in a median OS of no more than 13 months. While in the eastern countries, anticancer treatments are more aggressive: surgical resection, RT and TACE are recommended for selected HCC patients with PVTT by Chinese, Japanese, South Korean, and Asia-Pacific clinical practice guidelines because of their promising survival outcomes in the clinical practice (31, 32). Although, combination of TACE and LEN has been demonstrated clinical benefit in the treatment of advanced HCC by our previous study, its efficacy against PVTT is limited and the prognosis of HCC patients with PVTT is still dismal (19). Therefore, novel therapy modality including efficient local approach against PVTT should attach great importance.

The potential benefits of applying RT to the treatment of PVTT has been reported by previous studies (24, 25, 33–35), and one of the primary indications for RT is the macrovascular invasion. Yoon et al. reported in a randomized clinical trial that patients with HCC showing macroscopic vascular invasion in the TACE-RT group had a significantly higher radiologic response rate than the sorafenib group at 24 weeks (33.3% *vs.* 2.2%, *p*< 0.001), a significantly longer median time to progression (31.0 *vs.* 11.7 weeks, *p*< 0.001), and significantly longer OS (55.0 *vs.* 43.0 weeks, *p*= 0.040) (25). As in the current study, consistent results have been described that adding RT to TACE and LEN could further prolong the median OS and PFS to 22.8 and 12.8 months, respectively. The possible rationale for this triple combination therapy might be attributed to the quick reduction in tumor thrombus volume through radiation, which may relieve portal blood flow, allowing the maintenance of liver function, limiting intrahepatic tumor spread, and thereby allowing additional TACE (36, 37).Conversely, failure outside the radiation field could be complement by combining TACE and LEN treatment. Other combination therapy regimens are also being explored in advanced HCC. Recently, the CHANCE001 trial (38) demonstrated that combining TACE with PD-(L)1 blockade and molecular targeted treatments could improve survival outcomes and tumor response in Chinese patients with advanced HCC. However, the efficacy of this combination therapy for HCC patients with PVTT remains unknown as the trial did not show subgroup analysis for this population.

In terms of safety, most adverse events in our study were mild to moderate and could be manageable, which was similar to previous studies that combined therapy was used to treat advanced HCC (24, 25). More than 90% of patients receiving TACE-RT treatment experienced any-grade adverse events and 12% were reported with serious adverse events reported by Yoon et al. And the most common toxicities≥ grade 3 included AST/ALT increase (13.3%), diarrhea (2.2%), abdominal pain (2.2%), and bilirubin increase (2.2%) (25). As in our study, hepatic toxicity was the most common adverse event in TACE-LEN group, announcing approximately 20% of ALT/AST increase. Toxicity profile was similar in the RT-TACE-LEN group to that of TACE-LEN, and adding RT did not significantly increase the incidences of serious adverse events. Given these findings, toxicities caused by RT-TACE-LEN seemed to be acceptable and tolerable.

Several limitations should be considered in the current study. First, it was a nonrandomized controlled trial involving only three medical centers in China. Second, the number of included patients was relatively small. Thus, larger-sample sized, more medical centers and randomized-designed clinical trials are warranted to confirm the clinical benefit of this triple combination therapy.

In conclusion, our study demonstrated that, for patients with HCC showing PVTT, the combination therapy of RT-TACE-LEN was well tolerated and provided significantly improved PFS and OS compared with TACE-LEN. This novel triple combination therapy may be a promising treatment for patients with advanced HCC showing PVTT. Further studies are needed to confirm our findings.

## Data availability statement

The raw data supporting the conclusions of this article will be made available by the authors, without undue reservation.

## Ethics statement

The studies involving humans were approved by institution’s Ethics Committee (approval number: [2020]247. The studies were conducted in accordance with the local legislation and institutional requirements. The participants provided their written informed consent to participate in this study.

## Author contributions

AD: Writing – original draft. MZ: Writing – original draft. ZZ: Writing – original draft. WF: Investigation, Writing – review & editing. ZW: Methodology, Writing – review & editing. YC: Conceptualization, Writing – review & editing. JT: Conceptualization, Writing – original draft. YZ: Data curation, Writing – review & editing. WZ: Conceptualization, Writing – review & editing. XH: Writing – original draft, Writing – review & editing. ZP: Writing – review & editing.
