# Supplementary material for: Efficacy of radiation plus transarterial chemoembolization and lenvatinib in hepatocellular carcinoma with portal vein tumor thrombus
Source: Front Oncol. 2023 Dec 19;13:1320818. doi: 10.3389/fonc.2023.1320818 (PMC10763235; doi:10.3389/fonc.2023.1320818)
Supplement: Supplementary file 1 [file DataSheet_1.docx]

**Supplementary Materials**

**Supplemental Figure**

**Supplementary Fig. 1 Study schema**

**
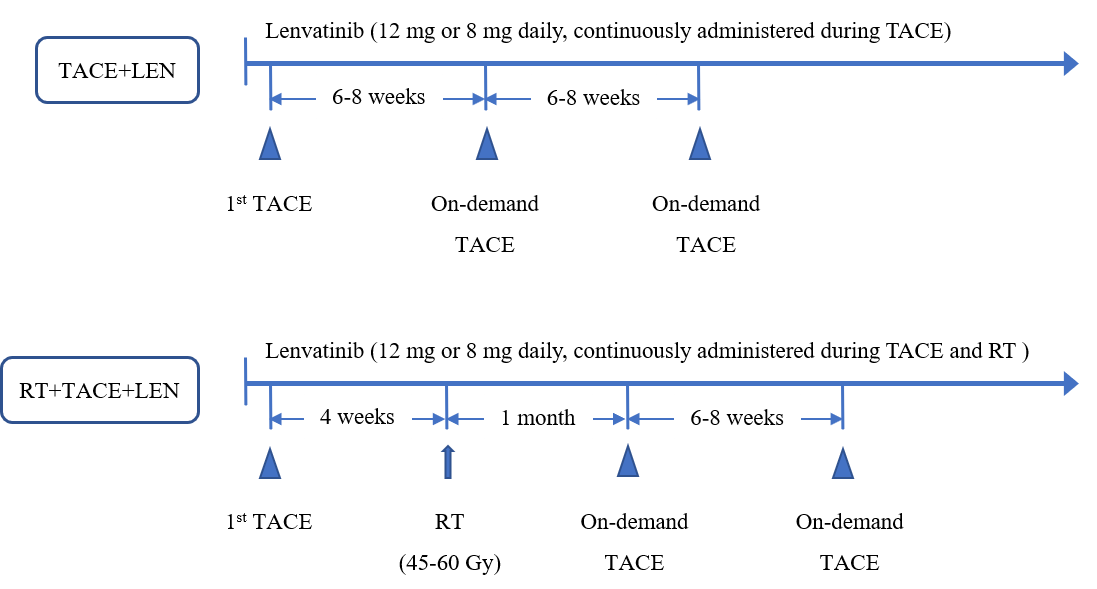
**

**Supplemental Tables**

**Supplementary Table. 1** **Short-term efficacy of the two treatment groups.**

| Variable | RT-TACE-LEN  *n*=51 (%) | TACE-LEN  *n*=51 (%) | *p* value |
| --- | --- | --- | --- |
| CR | 2 (3.9) | 1 (2.0) | 0.554 |
| PR | 27(52.9) | 25 (49.0) | 0.692 |
| SD | 18(35.3) | 21 (41.1) | 0.541 |
| PD | 4 (7.8) | 5 (9.8) | 0.727 |
| ORR | 29 (56.9) | 26 (51.0) | 0.551 |
| DCR | 47(92.1) | 46 (90.2) | 0.727 |

Abbreviations: RT, radiation; TACE, transarterial chemoembolization; LEN, lenvatinib; CR, complete response; PR, partial response; SD, stable disease; PD, progressive disease; ORR, objective response rate; DCR, disease control rate.

Note: The short-term efficacy was evaluated at 3 months after the completion of the last TACE in each group, or at 3 months after the completion of RT if no TACE was performed after RT.

**Supplementary Table. 2 Numbers of re-TACE, dTACE and cTACE.**

| Variable | RT-TACE-LEN  No. | TACE-LEN  No. | *p* value |
| --- | --- | --- | --- |
| re-TACE, mean (range) | 2 (1-5) | 2 (1-5) | 0.554 |
| DEB-TACE | 85 | 86 | 0.999 |
| cTACE | 80 | 84 | 0.846 |

Abbreviations: RT, radiation; TACE, transarterial chemoembolization; LEN, lenvatinib; DEB-TACE, drug-eluting transcatheter arterial chemoembolization; cTACE, conventional transarterial chemoembolization.

**Supplementary Table. 3 Subsequent treatments after progression.**

| Subsequent treatment | RT-TACE-LEN group,  No. (%) (n=30) | TACE-LEN group,  No. (%) (n=45) |
| --- | --- | --- |
| Anti-PD-1 immunotherapy | 13 (43.3) | 18 (40.0) |
| Other targeted therapy | 10 (33.3) | 16 (35.6) |
| TACE | 3 (10.0) | 5 (11.1) |
| Systemic chemotherapy | 1 (3.3) | 2 (4.4) |
| Radiotherapy | 2 (6.7) | 2 (4.4) |
| Conservative treatment | 1 (3.3) | 2 (4.4) |

Abbreviations: RT, radiation; TACE, transarterial chemoembolization; LEN, lenvatinib; PD-1, Programmed cell death protein-1.
